# Supplementary material for: A programmable DNA roadblock system using dCas9 and multivalent target sites
Source: PLoS One. 2022 May 6;17(5):e0268099. doi: 10.1371/journal.pone.0268099 (PMC9075669; doi:10.1371/journal.pone.0268099)
Supplement: S1 File — (PDF) [file pone.0268099.s001.pdf]

A Programmable DNA Roadblock System Using dCas9 and Multivalent Target Sites V.2

2
Feb 15, 2022

priceal [1], ekmatozel [1], parzialest [1]
[1]Emmanuel College

1

dx.doi.org/10.17504/protocols.io.b445qyy6

Emmanuel College

parzialest

A protein roadblock forms when a protein binds DNA and hinders translocation of other DNA binding proteins. These roadblocks can have significant effects on gene expression and regulation as well as DNA binding. Experimental methods for studying the effects of such roadblocks often target endogenous sites or introduce non-variable specific sites into DNAs to create binding sites for artificially introduced protein roadblocks. In this work, we describe a method to create programmable roadblocks using dCas9, a cleavage deficient mutant of the CRISPR effector nuclease Cas9. The programmability allows us to custom design target sites in a synthetic gene intended for *in vitro* studies. These target sites can be coded with multivalency—in our case, internal restriction sites which can be used in validation studies to verify complete binding of the roadblock. We provide full protocols and sequences and demonstrate how to use the internal restriction sites to verify complete binding of the roadblock. We also provide example results of the effect of DNA roadblocks on the translocation of the restriction endonuclease NdeI, which searches for its cognate site using one dimensional diffusion along DNA.

DOI

dx.doi.org/10.17504/protocols.io.b445qyy6

https://www.emmanuel.edu/academics/our-faculty/allen-price.html

priceal , ekmatozel , parzialest 2022. A Programmable DNA Roadblock System Using dCas9 and Multivalent Target Sites. **protocols.io**
https://dx.doi.org/10.17504/protocols.io.b445qyy6
parzialest

Citation: priceal , ekmatozel , parzialest A Programmable DNA Roadblock System Using dCas9 and Multivalent Target Sites https://dx.doi.org/10.17504/protocols.io.b445qyy6

This is an open access protocol distributed under the terms of the **Creative Commons Attribution License** (https://creativecommons.org/licenses/by/4.0/), which permits unrestricted use, distribution, and reproduction in any medium,

sgRNA, RNA, cas9, CRISPR, dcas9, roadblock, PCR, EcoRI, BtgI, NdeI, restriction endonuclease, translocation, single molecule, DNA cleavage

protocol ,

Feb 15, 2022

Feb 15, 2022

58237

Step 1:

- Nuclease-free water
- Q5 Master Mix (New England Biolabs)
- Forward and Reverse primers (10 uM) (See below)
- synthetic template DNA (See below)
- Sterile microcentrifuge tubes and PCR tubes
- PCR thermocycler

The sequence of the synthetic template DNA used:

CCAACTTAAT CGCCTTGCGC CGCCAGCTGG CGTAATAGCG AAGAGGCCCG CACCGATCGC
CCTTCCCAAC GTTGCGCAG CCTGAATGGC GAATGGCGCT TTGCCTGGTA TCCTGCACCA
GGAGCGCTGC CGGAAAGCTG GCTGGAGTGC ATCTTCCTG AGGCCGATAC TGTCGTCGTC
CCCTCAAACT GGCAGATGCA CGGTTACGAT GCGCCCATCT ACACCAACGT GACCTATCCC
ATTACGGTCA ATCCGCCGTT TGTTCCCACC TAGAATCCGA CGGGTTGTTA CTCGCTCACA
TTTAATGTTG ATGAAAGCTG GCTACAGGAA GGCCAGACGC GAATTATTTT TGATGGCGTT
CCTATTGGTT AAAAAATGGATATCGATCCACGGATCCAGG TGCGAATTTT AACAAAATAT
TTACGGTTAC AATTTGGGTA TTTGCTTATA CAATCTTCCT GTTTTTGGGG CTTTTCTGAT
TATCAACCGG GGTACATATG AATTCCATGC TAGTTTTACG ATTACCGTTC TTCGTTTCTC
TTGTTTGCTC CAGACTCTCA GGCAATGACC TGATAGCCTT TGTAGATCTC TCAAAAATAG
GGATATCGATCCACGGATCC TGGTCAGCTA GAACGGTTGA ATATCATATT GATGGTGATT
TGACTGTCTC CGGCCTTTCT CACCCTTTTG AATCTTTACC TACACATTAC TCAGGCATTG
CAGGGAAAAT ATATGAGGGT TCTAAAAATT TTTATCCTTG CGTTGAAATA AAGGCTTCTC
CCGCAAAAGT ATTACAGGGT CATAATGTTT TTGGTACAAC CGATTTAGCT TTATGCTCTG
AGGCTTTATT GCTTAATTTT GCTAATTCTT TGCCTTGCCT GTATGATTTA TTGGATGTTA
ATGCTACTAC TATTAGTAGA ATTGATGCCA CCTTTTCAGC TCGCGCCCCA AATGAAAATA
TAGCTAAACA GGTTATTGAG CGAAATGTAT CTAATGGTCA

The forward primer sequence: 5’-CGCGTTGGCCGATTC-3’
The reverse primer sequence: 5’-AGGGTGAGAAAGGCC-3’.

Step 6:

- PCR product from Step 5
- Qiagen PCR clean up kit (with spin columns and all reagents)

Citation: priceal , ekmatozel , parzialest A Programmable DNA Roadblock System Using dCas9 and Multivalent Target Sites
https://dx.doi.org/10.17504/protocols.io.b445qyy6

This is an open access protocol distributed under the terms of the **Creative Commons Attribution License** (https://creativecommons.org/licenses/by/4.0/), which permits unrestricted use, distribution, and reproduction in any medium,

- Microcentrifuge tube for storage of purified DNA

Step 8:

- Purified DNA from Step 7
- Water or EB buffer from PCR cleanup kit
- Nanodrop spectrophotometer

Step 14:

- Sterile nuclease-free microfuge tubes
- Nuclease-free water
- EnGen 2X sgRNA Reaction Mix, *S. pyogenes*
- Target-specific DNA oligo (1 µM) (see below)
    - Create by adding 1 µL oligo into 99 µL RNase free water
- DTT (0.1 M)
- EnGen sgRNA Enzyme Mix

The sequence of the target specific DNA oligo:
TTCTAATACGACTCACTATAGGATATCGATCCACGGATCCGTTTTAGAGCTAGA

Step 20:

- RNA product from Synthesis protocol above
- Monarch RNA Cleanup Kit (50 µg)
- ≥ 95% Ethanol
- Microcentrifuge tube for storage of purified RNA

Step 22:

- Sterile nuclease-free microcentrifuge tubes
- Nuclease-free Water
- NEBuffer 3.1
- 500 nM sgRNA
    - Dilute product from sgRNA synthesis with nuclease-free water
- 1 µM dCas9, *S. pyogenes*
- Substrate DNA
- Glycerol

PCR Preparation

Citation: priceal , ekmatozel , parzialest A Programmable DNA Roadblock System Using dCas9 and Multivalent Target Sites
https://dx.doi.org/10.17504/protocols.io.b445qyy6

This is an open access protocol distributed under the terms of the **Creative Commons Attribution License** (https://creativecommons.org/licenses/by/4.0/), which permits unrestricted use, distribution, and reproduction in any medium,

1 Gather the materials used in Step 1 (See Materials section). Thaw components before use. Keep all on ice.

2 Dilute the primers for (See Materials) to 10 uM before using.

PCR reaction

3 Combine the components from Step 1 into PCR tube(s) in order, for the desired final volume. Use Table 1 for reference.

A	B	C	D	E	F
**Component**	**Stock Concentration**	**4x Amount**	**2x Amount**	**1x Amount**	**Final Concentration**
Nuclease-free Water	-	78 µL	39 µL	19 µL	-
Q5 Master Mix	2x	100 µL	50 µL	25 µL	1x
Forward primer (DnnnnF)	10 µM	10 µL	5 µL	2.5 µL	500 nM
Reverse primer (BnnnnR)	10 µM	10 µL	5 µL	2.5 µL	500 nM
Template DNA	4 µg/mL	2 µL	1 µL	1 µL	0.2 - 0.4 µg/mL
**Total Volume**		**200 µL**	**100 µL**	**50 µL**	

Table 1: Q5 Master Mix

4 Perform PCR using Table 2 for parameters

A	B	C
**Step**	**Temperature (°C)**	**Time (Seconds)**
Denaturation	98	30
Melt	98	10
Anneal	60	30
Extend	72	30
Final Extend	72	120
Infinite Hold	4	-

Table 2: PCR Protocol

Citation: priceal , ekmatozel , parzialest A Programmable DNA Roadblock System Using dCas9 and Multivalent Target Sites https://dx.doi.org/10.17504/protocols.io.b445qyy6

This is an open access protocol distributed under the terms of the **Creative Commons Attribution License** (https://creativecommons.org/licenses/by/4.0/), which permits unrestricted use, distribution, and reproduction in any medium,

5 Advance to purification steps

Purification of PCR Product

6 Gather the materials used in Step 6 (See Materials section).

7 Perform Qiagen PCR purification kit by following the printed protocol inside the kit. Make sure pure ethanol (200 Proof) has been added to the correct buffer.

For 1x volume (50 uL total), you must use a blank centrifuge tube with water to counterbalance the spin column.

For 2x volume (100 uL total), use two spin columns and add 50 uL to each column. This will produce the same concentration as 1x protocol, but twice the volume (2x the amount of DNA).

For 4x volume (200 uL total), use two spin columns and add 100 uL (two PCR tubes) to each column. This will produce twice the concentration of the 1x protocol, and twice the volume (4x the amount of DNA).

Store on ice after purification.

Determination of DNA Concentration

8 Gather the materials used in Step 8 (See Materials section).

9 Use a nanodrop or similar UV absorbance instrument for measurements.

10 Blank with Buffer EB or dd H2O, depending on how you elute in your PCR purification kit.

11 Collect concentrations 2 times each.

12 Take the average of your 2 readings for each DNA sample.

Citation: priceal , ekmatozel , parzialest A Programmable DNA Roadblock System Using dCas9 and Multivalent Target Sites https://dx.doi.org/10.17504/protocols.io.b445qyy6

This is an open access protocol distributed under the terms of the **Creative Commons Attribution License** (https://creativecommons.org/licenses/by/4.0/), which permits unrestricted use, distribution, and reproduction in any medium,

13 Convert ng/uL to nM using the formula below. Record both ng/uL and nM numbers.

Concentration [nM] = 1.62 × Concentration [ng/uL]

Preparation of sgRNA

14 Gather the materials used in Step 14 (See Materials section).

15 Thaw EnGen 2X sgRNA Reaction Mix, *S. pyogenes*, and the customer-supplied target-specific oligo (1 µM). Mix and pulse each component in microfuge prior to use for 10 seconds. Store enzyme mix on ice.

16 Assemble the reaction at room temperature in the order listed below. Add the enzyme last to each reaction.

A	B
**Reagent**	**Amount**
Nuclease-free Water	2 µL
EnGen 2X sgRNA Reaction Mix, S. pyogenes	10 µL
Target-specific DNA oligo (1 µM)	5 µL
DTT (0.1 M)	1 µL
EnGen sgRNA Enzyme Mix	2 µL
**Total Volume**	**20 µL**

Table 4: EnGen 2X sgRNA Reaction Mix

17 Mix thoroughly and pulse-spin in a microfuge for 10 seconds. Incubate at 37°C for 30 minutes.

18 Transfer reaction to ice.

Citation: priceal , ekmatozel , parzialest A Programmable DNA Roadblock System Using dCas9 and Multivalent Target Sites https://dx.doi.org/10.17504/protocols.io.b445qyy6

This is an open access protocol distributed under the terms of the **Creative Commons Attribution License** (https://creativecommons.org/licenses/by/4.0/), which permits unrestricted use, distribution, and reproduction in any medium,

19 For DNase treatment, bring volume to 50 µL by adding 30 µL of nuclease-free water. Add 2 µL of DNase I, mix and incubate at 37°C for 15 minutes.

20 Gather the materials used in Step 20 (See Materials section).

21 Use Monarch RNA Cleanup Kit (50 µg) or similar RNA purification kit. From here, follow the protocol printed inside of the kit. Ensure that ethanol has been added to the correct buffer.

Activation of dCas9 and Binding to DNA

22 Gather the materials used in Step 22 (See Materials section).

23 Thaw NEBuffer 3.1, 500 nM sgRNA, and 1 µM dCas9. Mix and pulse each component in microfuge prior to use. Store tubes on ice.

24 Assemble the reaction below, in order, at room temperature. The final concentration of both the dCas9 and sgRNA should be 60 nM.

A	B
**Reagent**	**Amount**
Nuclease-free Water	19.14 µL
NEBuffer 3.1	3 µL
500 nM sgRNA	3.24 µL
1 µM dCas9, S. pyogenes	1.62 µL
**Total Volume**	**27 µL**

Table 5: dCas9 System Reaction Mix

25 Mix thoroughly by pipetting mixture up and down gently. Pulse-spin in a microfuge. Incubate at 25°C for 10 minutes.

Citation: priceal , ekmatozel , parzialest A Programmable DNA Roadblock System Using dCas9 and Multivalent Target Sites https://dx.doi.org/10.17504/protocols.io.b445qyy6

This is an open access protocol distributed under the terms of the **Creative Commons Attribution License** (https://creativecommons.org/licenses/by/4.0/), which permits unrestricted use, distribution, and reproduction in any medium,

26 Add enough DNA to reaction tube so that the final concentration of DNA is 3 nM. The ratio of dCas9:sgRNA:DNA should be 20:20:1.
- Ex. Add 1.12 µL of 75 nM stock DNA to reaction tube

27 Mix thoroughly by pipetting mixture up and down gently. Pulse-spin in a microfuge. Incubate at 37°C for 15 minutes.

28 Transfer reaction to ice.

29 Add enough glycerol to tube so that it is equal to total reaction volume. Mix thoroughly by pipetting mixture up and down.
- Ex. Add 28.12 µL glycerol to 28.12 µL reaction mixture

30 Roadblocks are now bound to programmed sites on DNA. Store in freezer.

Citation: priceal , ekmatozel , parzialest A Programmable DNA Roadblock System Using dCas9 and Multivalent Target Sites https://dx.doi.org/10.17504/protocols.io.b445qyy6

This is an open access protocol distributed under the terms of the **Creative Commons Attribution License** (https://creativecommons.org/licenses/by/4.0/), which permits unrestricted use, distribution, and reproduction in any medium,
