# Supplementary material for: A programmable DNA roadblock system using dCas9 and multivalent target sites
Source: PLoS One. 2022 May 6;17(5):e0268099. doi: 10.1371/journal.pone.0268099 (PMC9075669; doi:10.1371/journal.pone.0268099)

Using dCas9 and multivalent target sites as a programmable DNA roadblock system

Authors:
Emily K. Matozel, Stephen Parziale, Allen C. Price

Original full image of gel shown in Fig. 1 in manuscript.

Lane 1: DNA ladder.
Lane X1: irrelevant sample (not described in manuscript)
Lane X2: irrelevant sample (not described in manuscript)
Lane 2: DNA cleaved with Cas9 (as described in manuscript)
Lane 3: DNA without Cas9 (as described in manuscript)

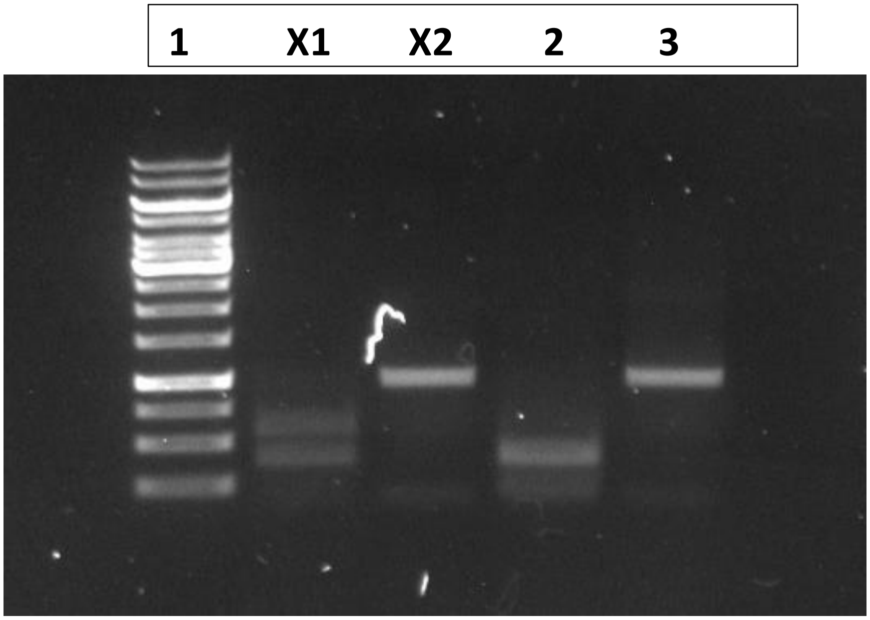

Supplement: S1 Fig — (PDF) [file pone.0268099.s002.pdf]
