# Supplementary material for: A programmable DNA roadblock system using dCas9 and multivalent target sites
Source: PLoS One. 2022 May 6;17(5):e0268099. doi: 10.1371/journal.pone.0268099 (PMC9075669; doi:10.1371/journal.pone.0268099)
Supplement: Attachment — Submitted filename: Response to Reviewers.docx [file pone.0268099.s003.docx]

**Dear Editor,**

**We thank you and the reviewers for the thorough and thoughtful reading of the manuscript. We have complied with all suggested revisions, which has improved the manuscript. We have also complied with the editorial suggestions specifically mentioned below. We believe this addresses all issues found with our original submission. Our responses are in blue.**

**Sincerely,**

**Allen Price**

**Editorial comments:**

We note that you have provided funding information that is not currently declared in your Funding Statement. However, funding information should not appear in the Acknowledgments section or other areas of your manuscript. We will only publish funding information present in the Funding Statement section of the online submission form. 
Please remove any funding-related text from the manuscript and let us know how you would like to update your Funding Statement. Currently, your Funding Statement reads as follows:

"ACP received grant # MCB-2120878 from the National Science Foundation ([www.nsf.gov](http://www.nsf.gov)). The funders have not had and will not have a role in study design, data collection and analysis, decision to publish, or preparation of the manuscript."

Please include your amended statements within your cover letter; we will change the online submission form on your behalf.

We have removed the grant reference in the Acknowledgement section. There are no changes to be made to the funding statement.

If applicable, we recommend that you deposit your laboratory protocols in protocols.io to enhance the reproducibility of your results. Protocols.io assigns your protocol its own identifier (DOI) so that it can be cited independently in the future.

We have done so. The protocol can be found at

<https://www.protocols.io/view/a-programmable-dna-roadblock-system-using-dcas9-an-36wgq422kvk5/v2>

While revising your submission, please upload your figure files to the Preflight Analysis and Conversion Engine (PACE) digital diagnostic tool, <https://pacev2.apexcovantage.com/>. PACE helps ensure that figures meet PLOS requirements.

We have uploaded all figures to PACE and are submitting the PACE adjusted figures.

**Reviewers' comments:**
**Reviewer #1:**
In this manuscript, Matozel et al describe a protocol to create in vitro roadblocks using deadcas9. This is an important area to study DNA protein barrier-related DNA metabolism including DNA repair and transcription. I have a few comments:

(1) In the Introduction, please cite novel work from Scully lab where they used bacteria system Tus/Ter to create DNA- protein block in a mammalian system and studied how DNA translocase protein FANCM regulate repair pathway choice at DNA protein barrier (Panday et al, Molecular Cell 2021). Please also cite the work from Natalia Y Tretyakova lab on using 5- Formylcytosine mediated DNA- Protein block Shaofei Ji et al Nucleic Acids research 2018. Also Goro Doi et al Nucleic acids research 2021 where they used dcas9 to impair fork progression.

We have included the requested references.

(2) Optional: Author Could use Chromatin Immunoprecipitation (CHIP) to study the specificity of roadblocks. If authors cannot do that experiment, please mention this in the discussion section as another way to confirm specificity.

We have added a short discussion section at the end of the manuscript and discussed extensions of our method to *in vivo* work, including the use of ChIP.

(3) Please mention the Sg sequence and highlight PAM sequence.

All sequences are included in the protocols.io website, as well as in the S1 file included. The sgRNA sequence is in the methods section, step 14, and the substrate DNA is in step 1. The PAM sequence is immediately adjacent to the 20 bp target sequence which is underlined in the substrate sequence.

**Reviewer #2:**

In this work, Matozel et al describe a detailed protocol to investigate programmable binding of dCas9 on double stranded DNA substrates and consequent blocking of diffusing/translocating molecules occupying the DNA. Understanding how multiple proteins simultaneously navigate/process DNA is an important question, and the use of a programmable roadblock as a model system lends itself to future investigation. The protocols are detailed and the accompanying experiments are robust, and their interpretation is sensible. The manuscript is well written and is very easy to follow. Overall, this is work that would benefit others in the field to answer mechanistic questions in protein-DNA interactions.

I only have one major comment which is that all figures should be accompanied by 'schematics' of the experimental setup (even if they have been described elsewhere). Without these schematics, naive readers may not be able to sufficiently visualize the experiment, and contextualize the results.

We have included a schematic of the DNA tethers used in the validation experiments as a separate panel in figure 2.
